# Overexpression of Homer1b/c induces valproic acid resistance in epilepsy

**DOI:** 10.1111/cns.14008

**Published:** 2022-11-09

**Authors:** Yan Wang, Youbin Li, Guangfei Wang, Jinmiao Lu, Zhiping Li

**Affiliations:** ^1^ Key Laboratory of Tropical Translational Medicine of Ministry of Education, Hainan Key Laboratory for Research and Development of Tropical Herbs, Haikou Key Laboratory of Li Nationality Medicine School of Pharmacy, Hainan Medical University Haikou China; ^2^ Department of Pharmacy Children's Hospital of Fudan University Shanghai China

**Keywords:** efficacy, Homer1b/c, HT22, pentylenetetrazol, VPA

## Abstract

**Aims:**

Resistance to valproic acid (VPA) is a major challenge for epilepsy treatment. We aimed to explore the mechanism underlying this resistance.

**Methods:**

Pentylenetetrazol‐induced chronic epileptic rats were administered VPA (250 mg/Kg) for 14 days; rats with controlled seizure stages (seizure score^14th‐before^ ≤0) and latent time (latent time^14th‐before^ ≥0) were considered VPA‐responsive, while the others were considered nonresponsive. Differentially expressed genes (DEGs) between the VPA‐responsive and nonresponsive rat hippocampus transcriptomes were identified, and their functions were evaluated. The roles of postsynaptic density (PSD) and Homer1 were also determined. Furthermore, a subtype of Homer1 (Homer1b/c) was overexpressed or silenced in HT22 cells to determine its effect on VPA efficacy. Moreover, the membrane levels of mGluR1/5 directly bound to Homer1b/c were assessed.

**Results:**

Overall, 264 DEGs commonly enriched in the PSD between VPA‐responsive and nonresponsive rats. Among them, Homer1 was more highly expressed in the hippocampus of nonresponses compared to that of responses. Overexpression of Homer1b/c interrupted VPA efficacy by increasing reactive oxygen species production, lactate dehydrogenase release, and calcium content. Furthermore, it induced the overexpression of mGluR1 and mGluR5.

**Conclusion:**

Overexpression of Homer1b/c influenced VPA efficacy, revealing it could be a target to improve the efficacy of this treatment.

## Graphical abstract

Cells responsive to valproic acid (VPA) showed decreased expression of Homer1b/c, reactive oxygen species production, and LDH release. On the contrary, overexpression of Homer1b/c interrupted VPA efficacy, resulting in higher reactive oxygen species production, calcium content, and LDH release.

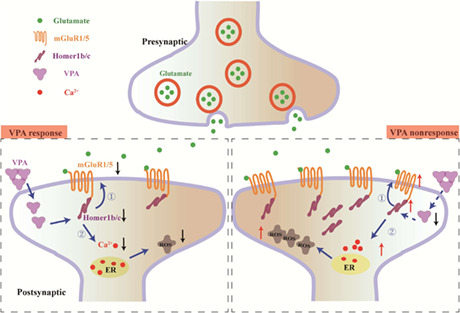

## 1 INTRODUCTION

Epilepsy is the most prevalent chronic neurological disorder, affecting approximately 70 million people worldwide.^1^ However, one‐third of the patients are refractory to antiepileptic drugs (AEDs) despite appropriate use.^2^ Although new AEDs, such as cannabidiol, have been developed, and the prevalence of refractory epilepsy remains unaffected.^3^, ^4^ Valproic acid (VPA) is an old AED that has been used in clinics for over 50 years due to its high efficacy and tolerability and is currently considered the first‐line therapy for absence epilepsy by the National Institute for Health and Care Excellence.^5^, ^6^ However, previous studies have shown that almost one‐third of the patients are nonresponsive to VPA.^7^, ^8^, ^9^ Interestingly, resistance to VPA is usually associated with genetic generalized forms of epilepsy.^10^, ^11^ Hence, it is imperative to elucidate the mechanism underlying VPA efficacy to improve the therapy of pharmacoresistant epilepsy.

Describing the pharmacokinetics and pharmacodynamics of a drug is the most common way to uncover the mechanisms underlying drug resistance; previously, using blood of samples of patients and epileptic rats, we demonstrated that the concentration of VPA was not associated with its efficacy.^12^, ^13^ Besides, it was also confirmed that VPA is not a substrate of multitransporters, such as P‐glycoprotein, multidrug resistant protein, and breast cancer resistance protein, nor does it induce the expression of these multitransporters in the brain.^14^, ^15^, ^16^, ^17^ Furthermore, several studies have demonstrated conflicting reports regarding the mutations or abnormal expression levels of VPA targets such as (𝛾‐aminobutyric acid, GABA) receptor, sodium channel, calcium channel, and N‐methyl‐D‐aspartic acid receptor (NMDAR) involved in the resistance of VPA,^18^, ^19^, ^20^, ^21^, ^22^, ^23^ undermining previous hypotheses involving multitransporters and targets as the key mechanism behind VPA resistance or efficacy. Presently, the mechanism of VPA inefficacy remains unclear.

Although some studies have explored the differences between the hippocampus of VPA‐nonresponsive patients and control subjects,^24^ there is a lack of accurate observation between VPA‐responsive and nonresponsive patients due to the limitations of the hippocampus from VPA‐responsive patients. To explore the molecular changes in the hippocampus of VPA‐responders and nonresponders, we previously established a pentylenetetrazol (PTZ)‐induced chronic rat model and administered VPA. VPA treatments showed limited efficacy, in line with the findings of previous studies using blood samples of patients.^13^ In the present study, we analyzed the differentially expressed genes (DEGs) and proteins of the hippocampus in VPA‐responsive and nonresponsive rats to explain the molecular mechanism of VPA resistance.

The postsynaptic receptors are the downstream elements of signal transmission that ultimately determine the transmission efficiency of excitatory synapses and might be the core of excitatory abnormalities of epileptic neural networks. The postsynaptic density (PSD) was highly expressed at postsynaptic neurons and was reported to modulate excitatory signal amplification and neurotransmitter clustering.^25^ Based on the DEGs analysis, we further determined the vital role of Homer1, a key component of the PSD associated with the etiology of epilepsy and related to VPA efficacy, in the glutamate‐induced HT22 cell line.

## 2 METHODS

### 2.1 Animals

Male Sprague–Dawley rats (postnatal day P7, certificate no. SCXK2014‐0011) were obtained from Tianqin Biotechnology Co., Ltd (Hunan, China). The rats were housed in standard plastic cages under controlled temperatures (25 ± 2°C) and illumination (12 h light/12 h dark) conditions and had access to a standard pelleted chow rat diet. Before any experimental manipulation, the rats underwent a one‐week initial adaptation period (P14, weight > 60 g). The study was approved by the Ethical Committee of Animal Care in Hainan Medical University. All efforts were made to reduce the number of animals used and minimize animal suffering and discomfort in accordance with the instructions of the Animal Research Reporting of In Vivo Experiment (ARRIVE) Guidelines 2.0.^26^

**FIGURE 1:**
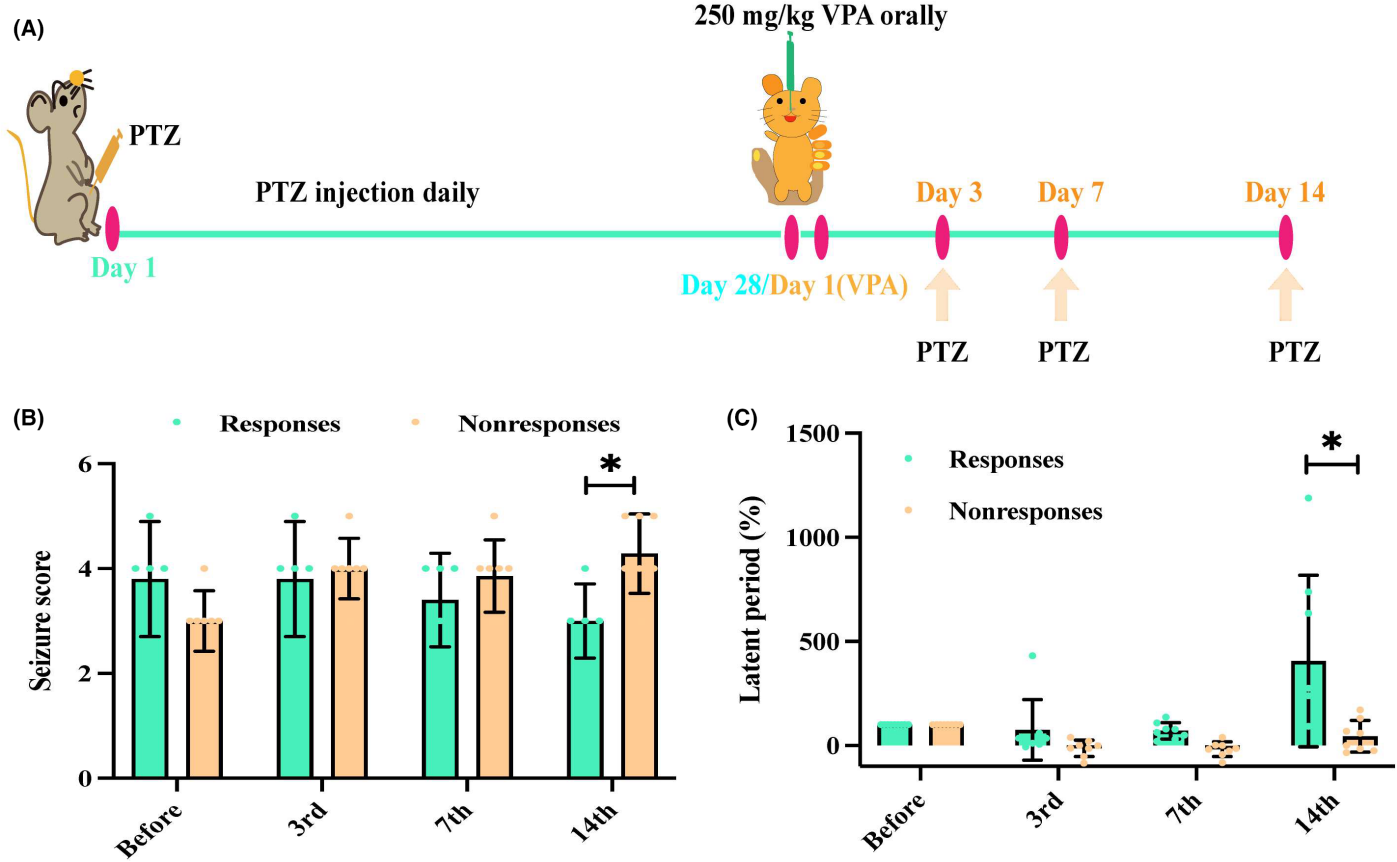
Behavior observation of responses and nonresponses. (A) Experimental procedure. Rats fully kindled with stage 3 seizures after three consecutive PTZ injections in 28 days were selected and orally administered with valproic acid (VPA) for the next 14 days. On the 3rd, 7th, and 14th days of VPA administration, rats were injected with PTZ to induce seizures to observe the effect of VPA. (B and C) Seizure score and latent period. After 14 days of VPA administration, epileptic rats resistant to VPA presented higher seizure score and lower latent period, compared with that of responsive rats. (Latent period (%): described by the formula (latent period ^(3rd, 7th, 14th)^ ‐ latent period^(before)^)/ latent period^(before)^).

A subconvulsive dose (35 mg/kg) of PTZ (Roche, Swiss) was administered by intraperitoneal (i.p.) injection for 28 days.^13^, ^27^ Fully kindled rats with stage 3 seizures after three consecutive PTZ injections were selected for the subsequent experiments. Then, those rats were orally administered with VPA (Depakine®, France) for 14 days. On the 3rd, 7th, and 14th days of VPA administration, rats were injected with PTZ to induce seizures to observe the effect of VPA (Figure 1A).

### 2.2 Seizure scoring

Seizures were scored according to Racine's criteria: stage 0: no response; stage 1: chewing and face twitching; stage 2: neck spasms and head nodding; stage 3: unilateral forelimb clonus and twitching; stage 4: rearing with bilateral forelimb clonus; and stage 5: generalized tonic–clonic seizures with loss of postural control.^28^ Latency to seizure onset was defined as the time elapsed between PTZ injection and the first observed seizure response.^29^ Rats with controlled seizure stages (seizure score^14th‐before^ ≤0) and latent time (latent time^14th‐before^ ≥0) were considered VPA‐responsive, while the others were considered nonresponsive.^13^

### 2.3 Transcriptome sequencing and molecular functions of the DEGs

Hippocampi from VPA‐responsive and nonresponsive rats were collected in TRIzol (Invitrogen, USA) and stored at −80°C before being used. Total RNA was extracted according to the manufacturer's instructions. RNA with high quality (OD260/280 = 1.8 ~ 2.2, OD260/230 ≥ 2.0, RIN ≥ 6.5, 28 S:18 S ≥ 1.0) and concentration (>2 μg) was used to construct the sequencing library using an Illumina HiSeq xten/NovaSeq 6000 sequencer (Agilent, Germany) by Shanghai Majorbio Bio‐pharm Technology Co. Ltd. Student's *t*‐test was performed to calculate the *P*‐value of genes with a fold change higher than 1.5. Those with *p*‐values lower than 0.05 were defined as DEGs.

Subsequently, the functions of DEGs were analyzed using the web‐based annotation tool DAVID v6.7 (https://david‐d.ncifcrf.gov/summary.jsp) to perform Gene Ontology (GO) and Kyoto Encyclopedia of Genes and Genomes (KEGG) pathway enrichment analyses.^30^ *p*‐value <0.05 was used as the threshold value.

### 2.4 Quantitative real‐time PCR

The mRNA levels of *Homer1a* and *Homer1b/c* in responders and nonresponders were analyzed using qPCR. Specific primers for the selected genes were designed using Primer‐BLAST (http://www.ncbi.nlm.nih.gov/tools/primer‐blast). All samples were amplified in triplicates according to the manufacturers' instructions (MonAmp, China). The primers used were as follows: *GAPDH* forward primer (F): 3'‐GGCATCCTGGGCTACACT‐5', reverse primer (R): 3'‐CCACCACCCTGTTGCTGT‐5'; *Homer1a* (F): 3'‐CCAGAAAGTATCAATGGGACAGATG‐5', (R): 3'‐TGCTGAATTGAATGTGTACCTATGTG‐5'; *Homer1b/c* (F): 3'‐TCCGTCTAGCAGCCAAGC‐5', (R): 3'‐TCTGTTGACGGTATTTCCTGTT‐5'. The relative expression of *Homer1a* and *Homer1b/c* was calculated based on the average Ct values across samples using the 2⁻^∆Ct^ with the expression level of *GAPDH* as the reference. Briefly, the formula used was relative expression = 2^ [− (Ct gene of interest ‐ Ct *GAPDH*)].

### 2.5 Western blot

Hippocampi were lysed with a protein lysis buffer containing phosphatase inhibitor (Boster, China). Proteins were transferred to a polyvinyl difluoride membrane following polyacrylamide gel electrophoresis. Then, the membrane was blocked with 5% skim milk followed by Homer1 antibody (Proteintech, China) at 1:1000 dilution at 4°C overnight. The results were visualized using a chemiluminescence detection system (Bio‐rad Laboratories, Inc).

### 2.6 Electron microscopy

The hippocampal tissue was fixed with ice‐cold glutaraldehyde (2% in 0.1 M cacodylate buffer, pH 7.4) for more than 2 h and fixed with osmium (1%) at 4°C for 3 h. Then, the sections were dehydrated in increasing concentrations of alcohol, embedded in spurs resin, and hardened at 70°C for 48 h. An ultramicrotome (EM UC6, Leica, Germany) was used to prepare 70‐nm brain slices that were stained overnight in uranyl acetate‐lead citrate (3%). Slices were imaged using a transmission electron microscope (JEM1230, JEOL) using an image pixel size of 0.2 nm.^31^

### 2.7 Immunofluorescence microscopy

Rats were deeply anesthetized and perfused via the ascending aorta with 0.9% NaCl solution, followed by 10% w/v buffered formalin solution. Slices were applied to perform immunofluorescence staining for Homer1b/c and mGluR5. Briefly, the sections were first incubated for 30 min in 5% bovine serum albumin in phosphate‐buffered saline (PBS) to block nonspecific binding and then incubated overnight at 4°C with mouse monoclonal antibody against Homer1b/c (1:100, Santa Cruz, USA) and mGluR5 (1:100, Invitrogen, USA). After washing with PBS, they were stained with 4′, 6‐diamidino‐2‐phenylindole (DAPI, Boster, China) for 5 min. A magnification microscope (Zeiss X‐Cite, German) was used to observe the stained cells. Figures were processed using the Image J software (1.52a, Wayne Rasband).

### 2.8 Cell culture and transfection

The murine hippocampal cell line, HT22 was purchased from BNCC (Bena Culture Collection, China). Cells were grown in high glucose Dulbecco's modified Eagle's medium (DMEM; Gibco) supplemented with 10% fetal bovine serum (CLARK) and streptomycin/penicillin (Biosharp, China) in a humidified incubator with 5% CO_2_ at 37°C.

Homer1b/c overexpression plasmid and siRNA were designed and manufactured by Shanghai Genechem Co., Ltd. (Shanghai, China) and HANBI Co., Ltd. (Shanghai, China), respectively. HT22 cells were transfected using Lipofectamine 2000 (Invitrogen) according to the manufacturer's instructions. Experiments using transfected cells were performed 24 h after transfection.

### 2.9 Cell viability assay

Cell viability was analyzed using Cell Counting Kit‐8 (CCK8, Biosharp, China). Briefly, cells were seeded and cultured into 96‐well microplates at a density of 1 × 10^4^ cells/well, using 100 μl of the medium. Then, the cells were treated with various concentration of glutamate (5, 10, 20, and 40 mM; Sigma) or VPA (0.01, 0.1, 1, and 10 mM; Sigma) for 24 h. Then, each well was incubated with 20 μl of CCK8 reagent for 30 min. The absorbance was measured at 450 nm using a microplate reader (Spectra MAX 190, Molecular Devices), using wells without cells as blanks. The proliferation of cells was evaluated using the measured absorbance according to a previously described method.^32^

### 2.10 Measurement of reactive oxygen species generation and calcium content

Intracellular ROS levels and calcium content were measured using 2', 7'‐dichlorofluorescin diacetate (H2DCFDA, Sigma) and Fura‐2 AM (Beyotime, China), respectively. Cells were exposed to glutamate alone or with VPA and then incubated with 10 μM H2DCFDA or 2 μM Fura‐2 AM for 30 min. The fluorescent intensity was used as an indicator of intracellular ROS level and calcium content using flow cytometry (NovoCyte, Agilent) with FITC or AmCyan channel. Fluorescent images were then obtained using a fluorescent microscope (Zeiss X‐Cite, Germany).

### 2.11 Lactate dehydrogenase (LDH) assay

A diagnostic kit was used to detect cellular LDH release into the culture medium following the manufacturer's instructions (Jiancheng Bioengineering Institute, China). The activity of LDH was calculated by measuring the absorbance at 440 nm using a microplate reader. Background absorbance from culture media that was not used for cell cultures was subtracted from all absorbance measurements.

### 2.12 Flow cytometry

Flow cytometry was applied to detect the expression levels of mGluR1. Briefly, HT22 cells were stained with 1 μg anti‐mGluR1(Boster, China) antibody for 30 min at 4°C. Then, the cells were washed with PBS thrice and incubated with a secondary APC‐conjugated antibody (Abcam) for 30 min at 4°C. The samples were washed with PBS and examined using a flow cytometer (NovoCyte, Agilent).

### 2.13 Statistical analysis

Data were expressed as the mean ± standard deviation (SD). Normal distribution test was performed using the Shapiro–Wilk test. Data followed a normal distribution and were analyzed using Student's *t*‐test for two groups and one‐way analysis of variance (ANOVA) for multiple groups. Otherwise, data were analyzed using the Kruskal–Wallis test. Results were considered significant at *p* < 0.05. Statistical analysis and generation of figures were performed using GraphPad Prism (Version 9.0.0) and R language (3.5.3).

## 3 RESULTS

### 3.1 Seizure behavior among VPA‐responsive and nonresponsive rats

After 14 days of VPA administration, epileptic rats resistant to VPA presented higher seizure scores (4.29 ± 0.76) and lower latent period (29.25 ± 51.24 s), compared with that of responsive rats (seizure score: 3.00 ± 0.71, latent period: 521.39 ± 500.89 s) (Figure 1B, C).

### 3.2 Postsynaptic density involved in the resistance to VPA

**FIGURE 2:**
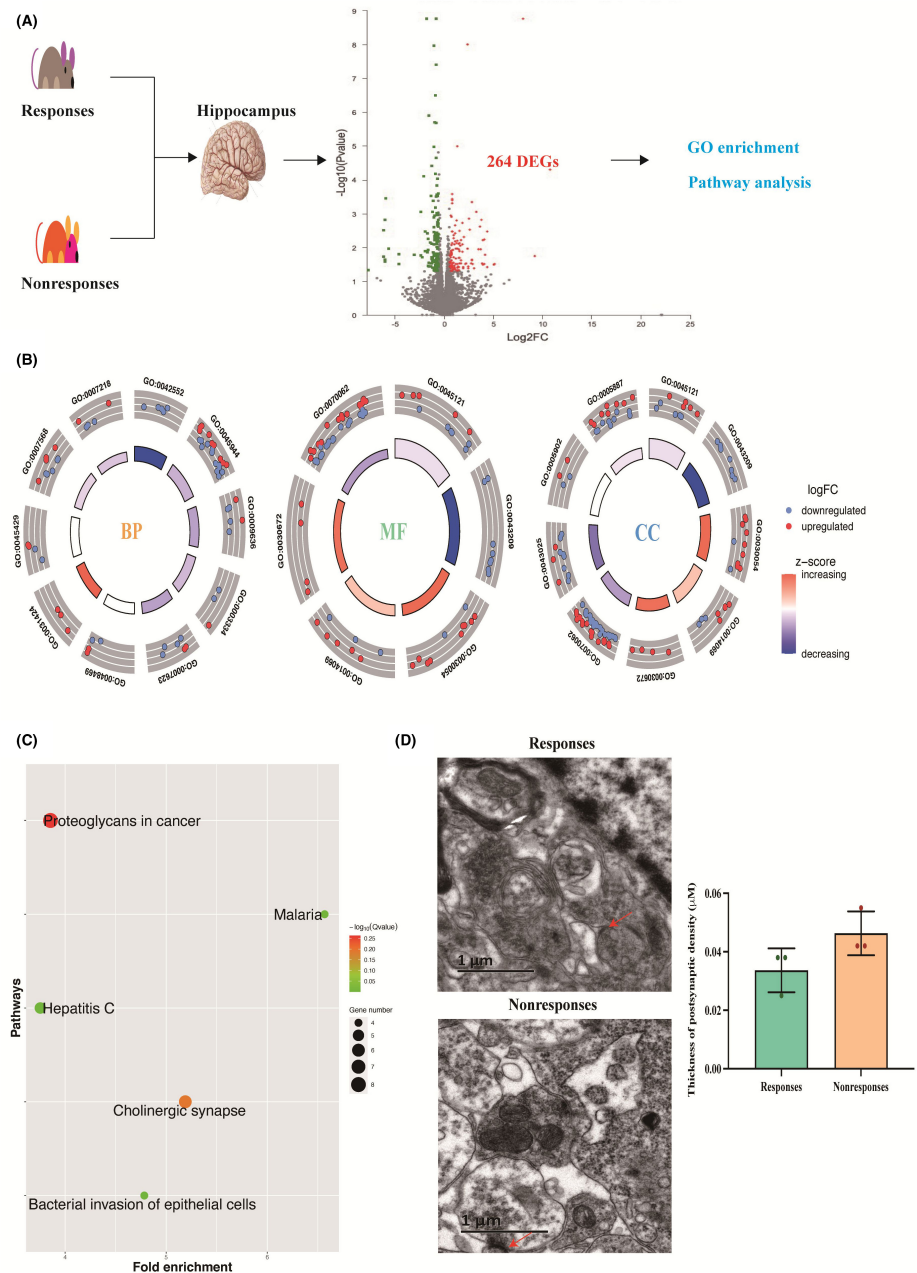
Transcriptome expression between VPA‐responsive and nonresponsive rats. (A) Procedure of transcriptome analysis. There were 264 differentially expressed genes (DEGs) in responsive and nonresponsive rats (green point represents downregulated genes, while red point represents upregulated genes). (B) GO enrichment. The 264 DEGs were enriched in the biological process (BP), cellular component (CC), and molecular function (MF) terms. Higher histogram in the inner circles represented lower *p*‐adjusted values, while, outer circles with red and blue indicated upregulated and downregulated DEGs. (C) Pathway analysis. Size of circles represented the number of gene enriched in the pathway, while, color varied from green to red indicated values of *p*‐adjusted from 0.05 to 0.25. (D) The thickness of PSD in CA1 region of VPA‐administered rats. Nonresponsive rats showed slightly higher thickness of PSD than that in responsive rats. (The red arrow represents the PSD, each group with *n* = 3).

To explore the differences in gene expression levels between responsive and nonresponsive rats, the mRNA expression levels in the hippocampus were measured. Overall, 264 genes presented a *p* value <0.05 and fold change >1.5 were defined as DEGs (Figure 2A). GO enrichment analyses indicated that these genes were enriched in the Biological Process (BP) terms—RNA polymerase 2 promoter, positive regulation of cell proliferation, and aging; they were enriched in the Molecular Function (MF) terms—structural molecule activity, Rab GTPase binding, and actin filament binding; and in the cellular component (CC)—neuronal cell body, cell junction, membrane raft, and PSD (Figure 2B and Table S1). Finally, KEGG enrichment analysis indicated that they were enriched in bacterial invasion of epithelial cells, malaria, hepatitis C, cholinergic synapse, and proteoglycans in cancer (Figure 2C).

To explore the role of PSD on the development of resistance to VPA, the thickness of PSD was assessed using electron microscopy. The thickness of PSD in nonresponses (463.3 ± 7.5 nm) was slightly higher than that in responses (336.7 ± 7.5 nm, Figure 2D).

### 3.3 The PSD (Homer1b/c) involved in the resistance to VPA

**FIGURE 3:**
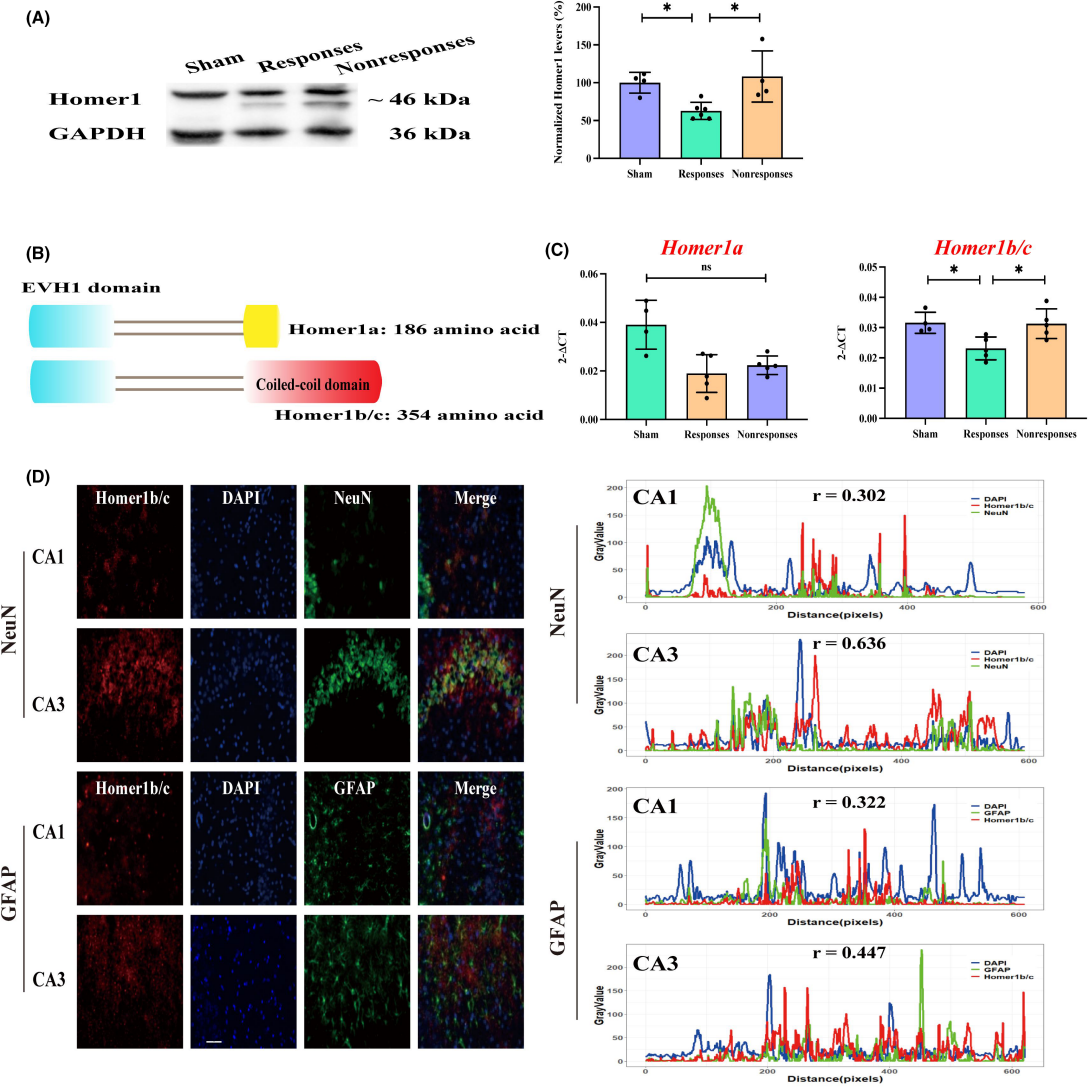
Homer1b/c associated with VPA resistance. (A) Protein expression of Homer1 in the hippocampi of sham (administered with saline), responsive, and nonresponsive rats. (B) Schematic figures of Homer1a and Homer1b/c. Homer1a (short splicing form) and Homer1b/c (long form) share a common enabled/vasodilator‐stimulated phosphoprotein homology (EVH1) domain; however, Homer1b/c contains an additional coiled‐coil domain. (C) mRNA expression of *Homer1a* and *Homer1b/c* in the hippocampus. VPA‐responsive rats showed lower expression of *Homer1b/c*, compared with that in sham and nonresponsive rats. However, there was no difference in *Homer1a* (D) Representative colocalization images and correlation analysis of Homer1b/c, NeuN (neurons), and GFAP (astrocytes) in the CA1 and CA3 regions of nonresponsive rats. Homer1b/c was mainly expressed in neurons of the CA3 region (overlap coefficient *r* = 0.636), and only slightly expressed in astrocytes (*r* = 0.447). Bar scale = 20 μm.

Homer scaffolding proteins 1 (Homer1) is an evolutionarily conserved key component of the PSD. In the present study, it was shown that the protein expression levels of Homer1 in responsive rats were lower than that in sham rats and nonresponsive rats (62.73 ± 11.27 VS 100.00 ± 13.76, 108.30 ± 33.85) (Figure 3A). As Homer1a and Homer1b/c are the most abundant members of the Homer family in the brain (Figure 3B), we further determined their mRNA expression levels using qPCR. No difference was observed in *Homer1a* expression level among the sham, responsive, and nonresponsive groups. However, the mRNA expression level of *Homer1b/c* was significantly decreased in responsive rats than in sham and nonresponsive rats (Figure 3C).

To determine which cells expressed Homer1b/c, double immunohistochemistry was performed in the CA1 and CA3 regions of the hippocampus using the markers neuronal nuclei (NeuN) and glia fibrillary acidic protein (GFAP), which are specific for neurons and astrocytes, respectively. Homer1b/c was mainly expressed in neurons of the CA3 region (overlap coefficient *r* = 0.636) and only slightly expressed in astrocytes (*r* = 0.447, Figure 3D).

### 3.4 VPA inhibited glutamate‐induced injury in the murine neuronal cell line HT22

Recurrent seizures and aberrant neuronal discharges result in transient excessive or abnormal neuronal activity in the brain. The excessive release of glutamate in this situation leads to excitotoxicity and oxidative stress, which play a major role in the etiology of epilepsy.^33^, ^34^ Therefore, we applied a glutamate‐induced injury model to explore the effects of Homer1b/c on VPA efficacy.

**FIGURE 4:**
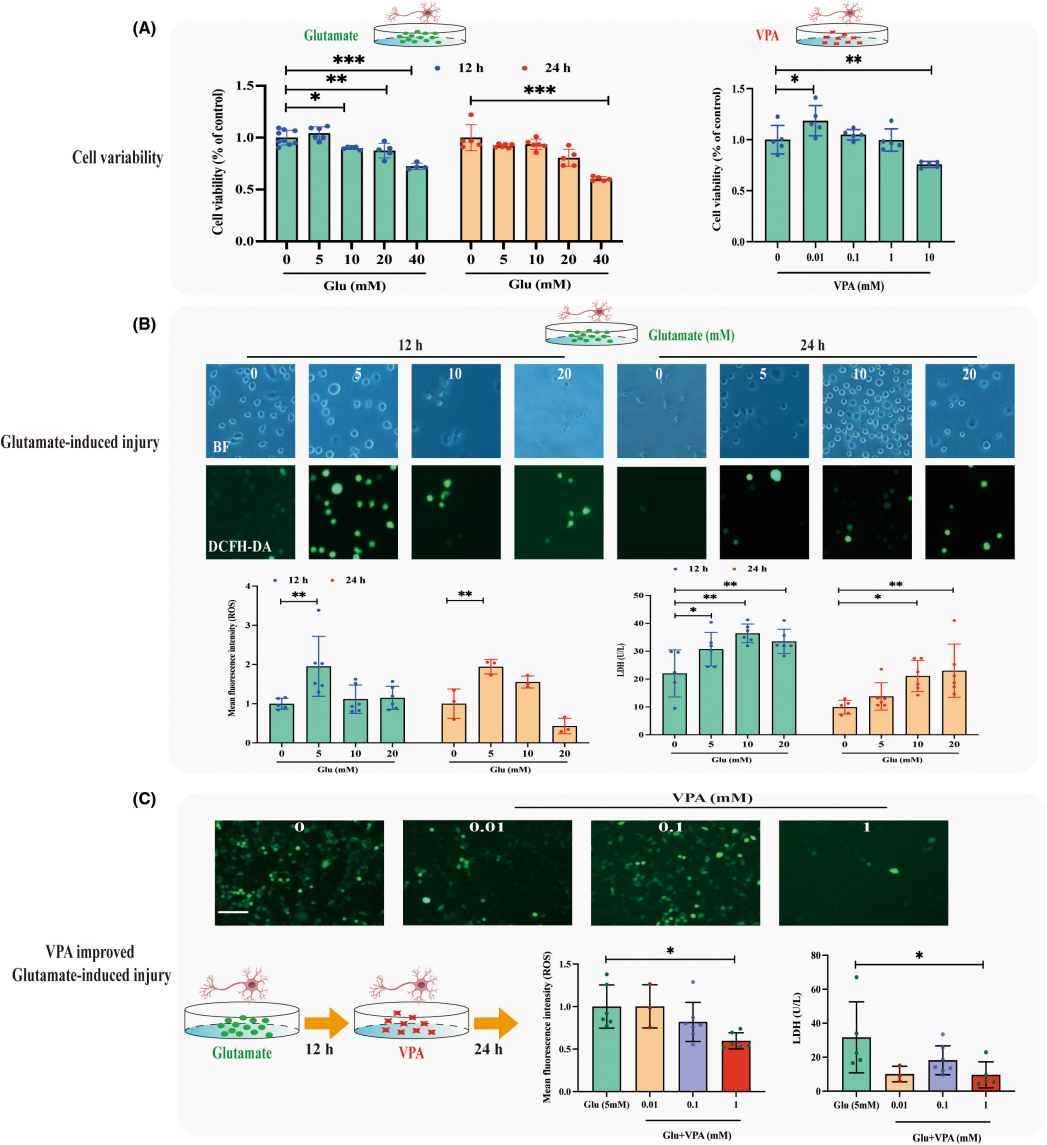
VPA inhibited glutamate‐induced injury of mouse neuron cell line HT22. (A) Cell viability of glutamate and VPA on HT22 cells. The administration of glutamate (40 mM for 12 or 24 h) or VPA (10 mM for 24 h) significantly inhibited the cell viability of HT22, compared with the control group. (B‐C) Effects of VPA on glutamate‐induced injury. Administration of 5 mM of glutamate for 12 h resulted in significantly higher mean fluorescence intensity of DCFH‐DA (indicator of reactive oxygen species, ROS) and LDH release simultaneously, compared with the control group (B), while further treatment with 1 mM of VPA for 24 h showed decreased ROS production and LDH release, compared with those without VPA (C), bar scale = 100 μm.

First, we evaluated the effects of different concentrations of glutamate or VPA on the viability of HT22 cells using a CCK8 assay. The results demonstrated that the administration of glutamate above 40 mM for 12 or 24 h significantly inhibited the cell viability of HT22, compared with the control group. Treatment with a high (10 mM) concentration of VPA for 24 h significantly inhibited the proliferation of HT22. (Figure 4A). Therefore, concentrations of glutamate (5, 10, 20 mM) and VPA (0.01, 0.1, 1 mM) were used in the further experiment.

Then, we measured glutamate‐induced injury by evaluating ROS production and LDH release. Administration of 5 mM of glutamate for 12 h resulted in significantly higher oxidative injury and LDH release simultaneously, compared with the control group (Figure 4B). Based on these results, the condition of 5 mM glutamate administration for 12 h was selected for the following experiments.

Cells preadministered with 5 mM glutamate for 12 h and further treated with 1 mM of VPA for 24 h showed decreased ROS production and LDH release, compared with those without VPA. However, other concentrations (0.01 and 0.1 mM) of VPA did not improve glutamate‐induced injury (Figure 4C).

### 3.5 Homer1b/c associated with the effect of VPA on glutamate‐treated HT22 cells

**FIGURE 5:**
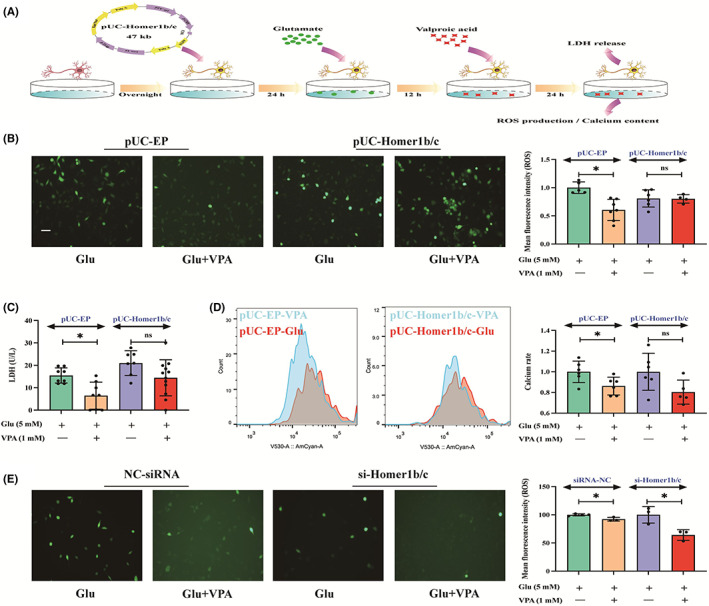
Homer1b/c associated with VPA efficacy in glutamate‐treated HT22 cells. (A) Experimental procedure. HT22 cells were transfected with plasmids encoding with *Homer1b/c* or siRNA to induce Homer1b/c overexpression or silence *Homer1b/c* for 24 h followed by glutamate administration for 12 h and VPA for 24 h, successively. (B‐D) Mean fluorescence intensity of ROS, LDH release, and calcium content in the empty‐plasmid (EP) and Homer1b/c overexpression group. In the Homer1b/c overexpression cells, 1 mM of VPA did not improve the ROS production, LDH release, and calcium content compared with glutamate‐induced cells. (E) ROS production in the control‐siRNA (NC‐siRNA) and si‐Homer1b/c group. VPA could inhibit the ROS production in the Homer1b/c‐knockdown HT22 cell. Bar scale = 100 μm.

To confirm the role of Homer1b/c on VPA efficacy in glutamate‐treated HT22 neurons, cells were transfected with plasmids encoding with *Homer1b/c* to induce Homer1b/c overexpression before glutamate treatment (Figure 5A). In the empty‐plasmid (EP) group, ROS production, LDH release, and calcium content in VPA‐treated neurons were significantly decreased, compared to those in glutamate‐treated cells. However, in the Homer1b/c overexpression cells, 1 mM of VPA did not improve the ROS production (Figure 5B), LDH release (Figure 5C), and calcium content (Figure 5D) compared with glutamate‐induced cells. On the contrary, VPA could inhibit ROS production in the Homer1b/c‐knockdown HT22 cell (Figure 5E).

### 3.6 Group I metabotropic glutamate receptors (mGluR1 and mGluR5) involved in the effect of Homer1b/c on the resistance of VPA

**FIGURE 6:**
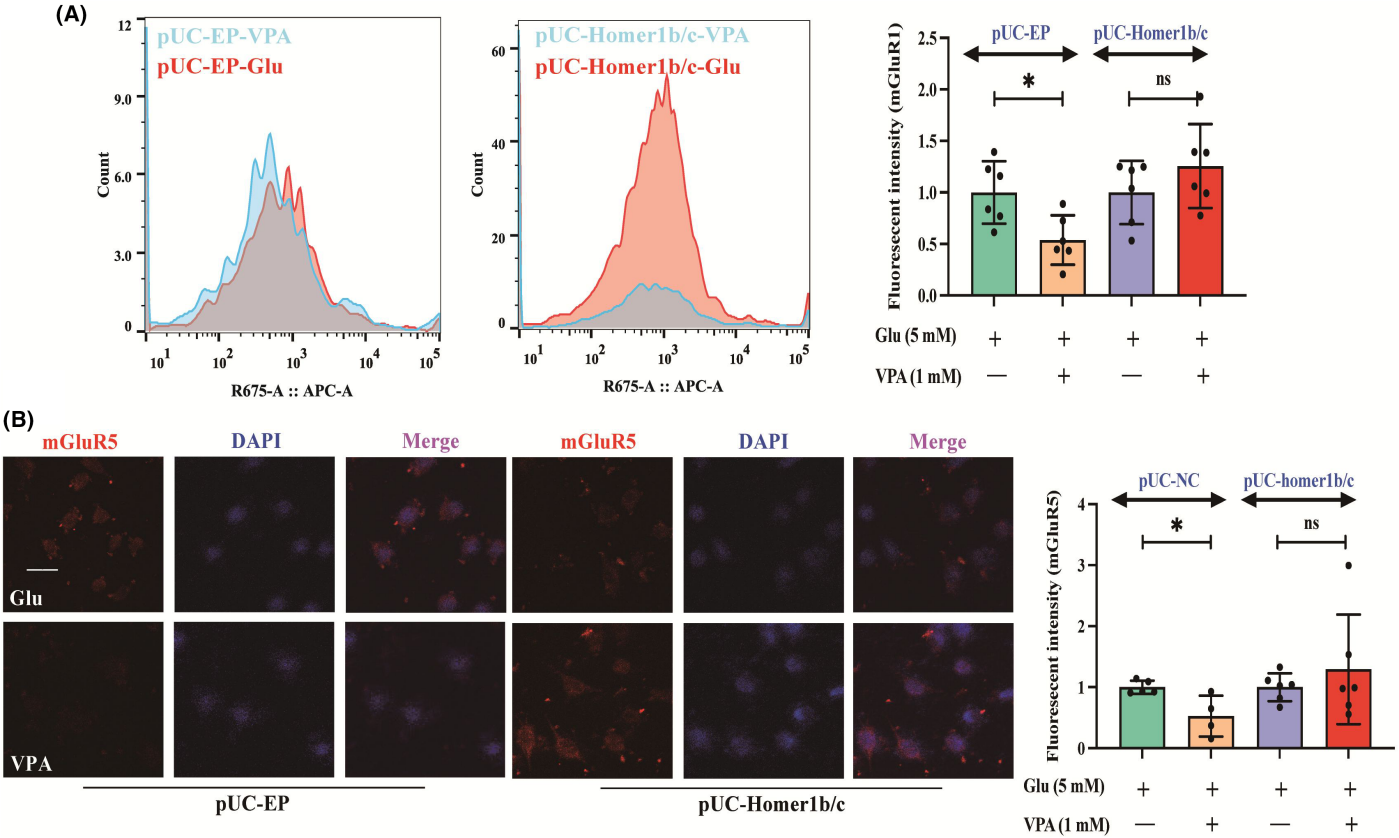
Overexpression of Homer1b/c interrupted the protein expression of mGluR1 and mGluR5 (A‐B) The expression of mGluR1 and mGluR5 in the HT22 cells. The expression of mGluR1 and mGluR5 in the EP cells was dramatically decreased in the VPA‐treated group compared with the glutamate‐treated group. However, administration of VPA to Homer1b/c‐overexpression HT22 cells did not induce any changes. Bar scale = 20 μm.

Homer1b/c is a postsynaptic protein that binds to mGluR1 and mGluR5.^35^, ^36^ Hence, we assessed the change in mGluR1 and mGluR5 expression levels after overexpressing Homer1b/c. The membrane expression of mGluR1 and mGluR5 in the EP cells was dramatically decreased in the VPA‐treated group compared with the glutamate‐treated group. However, administration of VPA to Homer1b/c‐overexpression HT22 cells did not induce any changes in expression levels of mGluR1 (Figure 6A) and mGluR5 (Figure 6B). These indicated that the overexpression of Homer1b/c tended to induce distribution of mGluR1 and mGluR5.

## 4 DISCUSSION

Although new AEDs, such as cannabidiol, have been applied to treat epilepsy, their efficacy for neither new‐onset nor chronic refractory epilepsy is better than that of older drugs.^4^, ^37^ VPA is the most commonly used old AED; however, 30% of epileptic patients tend to show resistance. To date, resistance to VPA is still a major challenge. In the present study, we identified 264 DEGs commonly enriched in PSD by analyzing the hippocampus of responsive and nonresponsive rats. The PSD is an architecture comprising specialized proteins responsible for mediating the response to converging synaptic input, and its abnormal function plays an important role in the pathogeny of epilepsy. Mutations in gephyrin, an essential core scaffolding protein, have been demonstrated to be involved in the regulation of inhibitory PSD associated with pharmacoresistant epilepsy.^38^ Furthermore, the alternative expression of the microtubule units (α‐ and β‐tubulin) and components (SH3 and multiple Ankyrin repeat domains 3, and postsynaptic density protein‐93) of PSD in excitatory neurons has been revealed in latent and chronic epilepsy.^39^, ^40^, ^41^

Homer scaffolding proteins (Homer1‐3) are evolutionarily conserved key components of the PSD. Homer 1 is highly expressed in the cortex^42^ and hippocampus,^43^ which are regions associated with the etiology of epilepsy. The two major splice variants of Homer1 are Homer1a (short splicing form) and Homer1b/c (long form) that share a common enabled/vasodilator‐stimulated phosphoprotein homology (EVH1) domain and a proline motif. Homer1b/c contains an additional coiled‐coil domain that allows the formation of homo‐ and heterocomplexes of Homer proteins. The lack of a coiled‐coil domain in Homer1a causes this isoform to act as a competitor to Homer1b/c, resulting in uncoupling of the interaction between Homer1b/c and NMDAR, α‐amino‐3hydroxy‐5‐methyl‐4‐isoxazole‐propionic acid receptor (AMPAR), or mGluRs.^44^ It has been demonstrated that Homer1a expression levels are increased during seizures, whereas it is downregulated during the chronic period of epilepsy.^45^ On the contrary, Homer1b/c was found to be unchanged during seizures, but upregulated during the chronic period.^46^ Furthermore, silencing Homer1b/c dramatically decreased the spike frequency and seizure score of mice.^47^ To the best of our knowledge, this is the first study reporting that Homer1b/c expression levels were higher in rats nonresponsive to VPA compared with responsive rats.

Glutamate, the predominant excitatory transmitter, has been reported to be reduced by VPA.^48^ In HT22 cells, administration of 1 mM VPA significantly prevented glutamate‐induced neuron injury by inhibiting ROS production, calcium content, and LDH release. This result is in line with those of the previous studies in which 1 mM VPA significantly ameliorated radiation‐ and hemin‐induced neuron injury.^49^, ^50^ However, as HT22 cells overexpress Homer1b/c, they were not responsive to VPA, confirming that the overexpression of Homer1b/c in neurons disrupted VPA efficacy.

Group I metabotropic glutamate receptors, such as mGluR1 and mGluR5, are the major targets of glutamate.^51^, ^52^ When glutamate binds to these receptors, the corresponding G proteins and cytoplasmic signaling pathways are activated.^53^ Increasing expression of mGluR1 and mGluR5 was found in the cortex of kindling rats and the hippocampus of pharmaco‐resistant temporal lobe epilepsy patients, respectively.^54^, ^55^, ^56^ Homer1b/c interacts with proline‐rich sequences of mGluR1 and mGluR5 through its EVH1 domain^57^ and alters localization of mGluR1 and mGluR5, redistributing them from soma to dendritic synaptic sites.^35^, ^58^ Our study revealed that overexpression of Homer1b/c induced the expression of mGluR1 and mGluR5 simultaneously. These results are consistent with those of a previous study, which demonstrated that transfecting cells with concentrations of Homer1b cDNA above 1.5 μg resulted in endoplasmic reticulum (ER) retention of mGluR5.^59^ Here, 2.0 μg of Homer1b/c were used to transfect HT22 cells. High expression of mGluR1 and mGluR5 has been reported to induce the clustering of inositol triphosphate 3 receptors in the ER, thereby resulting in intracellular calcium release.^60^ In line with this, we demonstrated high intracellular calcium release in the Homer1b/c‐overexpressing HT22 cells. Hence, we hypothesize that overexpression of Homer1b/c‐induced VPA resistance by increasing mGluR1 and mGluR5 levels in the plasma membrane of neurons and interrupting the ROS production and calcium release in the cytoplasm.

To determine whether increasing concentrations of VPA could lead to a more significant efficacy on glutamate‐treated Homer1b/c‐overexpressing HT22 cells, we augmented the VPA concentration to 2 and 4 mM. However, both those doses also failed to prevent glutamate‐induced cytotoxicity (data not shown). These results were in accordance with those of Hiroi et al. that demonstrated higher concentrations of VPA were not protective at 2 mM, but potentiated cytotoxicity at 3–5 mM.^61^ Hence, it suggested that increasing VPA concentration did not improve Homer1b/c induced resistance.

Biomarkers that predict drug‐resistant epilepsy make great sense for the treatment of epilepsy. Specially, CpG methylation, miR‐146, and miR‐223, which are easily measured in the blood, were revealed to predict drug‐resistant epilepsy.^62^, ^63^, ^64^ Homer1 was highly expressed in the brain, whole blood, and platelet; however, its mRNA expression in the hippocampus of rats was not comparable with that in whole blood, which restricted its ability to predict VPA efficacy^65^ and was one of the limitations of the present study. However, whether the reporting regulators of Homer1, such as miR‐1271,^66^ could be potential biomarkers to predict VPA efficacy needs further study. Considering the higher prevalence of active epilepsy in males than in females^67^ and the restricted utilization of VPA in females due to fetal malformation,^68^ we only used male rats in the present study. However, sexual dimorphism exists in brain microvessels,^69^ cerebral perfusion,^70^ brain mitochondrial metabolism,^71^ and brain injury outcomes. Lack of data in both males and females is another limitation of the current study. Hence, a confirmatory study should be done on female animals in the future.

## CONCLUSIONS

In summary, this study demonstrated that the overexpression of Homer1b/c disrupted VPA efficacy by increasing the expression of mGluR1/5 and induced ROS production, LDH release, and calcium content in the cytoplasm, suggesting that overexpression of Homer1b/c could elucidate the mechanism of VPA resistance to some extent.

## AUTHOR CONTRIBUTIONS

Yan Wang designed the experiments, performed the experiments, and wrote the manuscript. Youbin Li designed the experiments, analyzed the data, and revised the manuscript. Guangfei Wang and Jinmiao Lu analyzed the transcriptome data and drew figures. Zhiping Li designed the experiments and revised the manuscript.

## FUNDING INFORMATION

This work was supported by the National Natural Science Foundation of China (No. 81874325), Scientific Research Project of Science and Technology Commission of Shanghai Municipality (No. 19DZ1910703), Hainan Provincial Natural Science Foundation of China (Nos. 819QN224, 820QN265), Scientific Cultivation Foundation of Hainan Medical University (No. HYPY201909), and Epilepsy Research Science Innovation Group of Hainan Medical University (2022).

## CONFLICT OF INTEREST

The authors declare that they have no competing interest.

## Supporting information


TableS1

## Data Availability

The data that support the findings of this study are available from the corresponding author upon reasonable request.
