# Correction to “Overexpression of Homer1b/c induces valproic acid resistance in epilepsy”

**DOI:** 10.1111/cns.14785

**Published:** 2024-05-26

Wang Y, Li Y, Wang G, Lu J, Li Z. Overexpression of Homer1b/c induces valproic acid resistance in epilepsy. *CNS Neurosci Ther*. 2023;29:331‐343. doi:10.1111/cns.14008

In the section of funding information, we miswrote the number of funding (819QN265). The correct name should be presented as following:

This work was supported by the National Natural Science Foundation of China (No. 81874325), Scientific Research Project of Science and Technology Commission of Shanghai Municipality (No. 19DZ1910703), Hainan Provincial Natural Science Foundation of China (Nos. 819QN224, 820RC630), Scientific Cultivation Foundation of Hainan Medical University (No. HYPY201909), and Epilepsy Research Science Innovation Group of Hainan Medical University (2022).

We apologize for this error.

