# Supplementary material for: Overexpression of Homer1b/c induces valproic acid resistance in epilepsy
Source: CNS Neurosci Ther. 2022 Nov 9;29(1):331–43. doi: 10.1111/cns.14008 (PMC9804053; doi:10.1111/cns.14008)
Supplement: Supplementary file 1 — TableS1 [file CNS-29-331-s001.docx]

**Supplementary Table 1.** The detail information of GO enrichment.

| Category | GO No. | Term | Fold Enrichment | *P* Value |
| --- | --- | --- | --- | --- |
| BP | GO:0006682 | galactosylceramide biosynthetic process | 93.77 | 0.021 |
|  | GO:0032741 | positive regulation of interleukin-18 production | 46.89 | 0.042 |
|  | GO:0003334 | keratinocyte development | 20.09 | 0.009 |
|  | GO:0031424 | keratinization | 18.75 | 0.011 |
|  | GO:0003009 | skeletal muscle contraction | 9.38 | 0.040 |
|  | GO:0031069 | hair follicle morphogenesis | 9.07 | 0.043 |
|  | GO:0048469 | cell maturation | 8.72 | 0.011 |
|  | GO:0042552 | myelination | 8.68 | 0.003 |
|  | GO:0045429 | positive regulation of nitric oxide biosynthetic process | 8.34 | 0.012 |
|  | GO:0071320 | cellular response to cAMP | 4.87 | 0.049 |
|  | GO:0007218 | neuropeptide signaling pathway | 4.83 | 0.020 |
|  | GO:0009636 | response to toxic substance | 4.73 | 0.009 |
|  | GO:0007623 | circadian rhythm | 4.65 | 0.009 |
|  | GO:0007568 | aging | 2.68 | 0.019 |
|  | GO:0010628 | positive regulation of gene expression | 2.27 | 0.045 |
|  | GO:0008284 | positive regulation of cell proliferation | 2.05 | 0.042 |
|  | GO:0045893 | positive regulation of transcription, DNA-templated | 1.97 | 0.041 |
|  | GO:0045944 | positive regulation of transcription from RNA polymerase II promoter | 1.92 | 0.006 |
|  | GO:0055114 | oxidation-reduction process | 1.87 | 0.045 |
| CC | GO:0030672 | synaptic vesicle membrane | 6.44 | 0.024 |
|  | GO:0005902 | microvillus | 5.43 | 0.037 |
|  | GO:0043209 | myelin sheath | 3.98 | 0.004 |
|  | GO:0045121 | membrane raft | 3.93 | 0.001 |
|  | GO:0014069 | postsynaptic density | 3.19 | 0.013 |
|  | GO:0030054 | cell junction | 2.55 | 0.011 |
|  | GO:0043025 | neuronal cell body | 2.11 | 0.027 |
|  | GO:0005887 | integral component of plasma membrane | 1.71 | 0.039 |
|  | GO:0070062 | extracellular exosome | 1.40 | 0.025 |
| MF | GO:0019911 | structural constituent of myelin sheath | 27.30 | 0.005 |
|  | GO:0017137 | Rab GTPase binding | 4.33 | 0.012 |
|  | GO:0051015 | actin filament binding | 3.93 | 0.018 |
|  | GO:0001078 | transcriptional repressor activity, RNA polymerase II core promoter proximal region sequence-specific binding | 3.86 | 0.041 |
|  | GO:0005198 | structural molecule activity | 3.37 | 0.018 |
|  | GO:0043565 | sequence-specific DNA binding | 1.92 | 0.048 |
